# Surveillance of Dialysis Events: one-year experience at 33 outpatient hemodialysis centers in China

**DOI:** 10.1038/s41598-017-00302-9

**Published:** 2017-03-21

**Authors:** Hui Zhang, Liuyi Li, Huixue Jia, Yunxi Liu, Jianguo Wen, Anhua Wu, Qun Lu, Tieying Hou, Yun Yang, Huai Yang, Weiguang Li, Zhiyong Zong

**Affiliations:** 1 Department of Infection Control, West China Hospital, Sichuan University, Chengdu, China; 2 Department of Infection Control, First People’s Hospital of Beijing University, Beijing, China; 3 Department of Infection Control, Peoples Liberation Army General Hospital, Beijing, China; 4 Department of Infection Control, First Affiliated Hospital of Zhengzhou University, Zhengzhou, China; 5 Department of Infection Control, the first hospital affiliated to Xiangya Medical School, Changsha, China; 6 Department of Infection Control, the Second Affiliated Hospital of Zhejiang University School of Medicine, Hangzhou, China; 7 Department of Infection Control, Guangdong Provincial People’s Hospital, Guangzhou, China; 8 Department of Infection Control, First Hospital of Shanxi Medical University, Taiyuan, China; 9 Department of Infection Control, Guizhou Provincial People’s Hospital, Guizhou, China; 10 Department of Infection Control, Shandong Province Hospital, Jinan, China; 11 Center of Infectious Diseases, West China Hospital, Sichuan University, Chengdu, China

## Abstract

A multicenter prospective surveillance on dialysis events was carried in 33 dialysis centers in China. Maintenance hemodialysis (HD) outpatients who were dialyzed on the first two days of each month during 2014 were monitored for dialysis events and other infections. During the one-year period, 52,680 patient-months were monitored. Fistula and tunneled or non-tunneled central line were used for 73.70%, 15.70% and 8.85% of vascular access, respectively. There were 773 dialysis events occurred in 671 patients including 589 IV antimicrobial starts, 74 positive blood cultures and 110 local access site infections (LASI). The incidence of dialysis events was 1.47 per 100 patient-months. Among the 74 cases with bloodstream infection (BSI), 38 were access-related BSI (ARB) and there were therefore 148 cases with vascular-related infection (VAI; 38 ARB and 110 LASI). There were 740 cases (1.40 per 100 patient-months) with infections other than BSI and LASI, most (79.19%) of which were respiratory tract infections. For those with dialysis events, there were 425 cases (425/671, 63.34%) admitted to hospital and 12 cases of death (12/671, 1.79%). In conclusion, the surveillance revealed a relatively low incidence of dialysis events and the surveillance may be tailored to target those using central lines in resource-limited settings.

## Introduction

According to the data that Chinese Hospital Association collected from dialysis centers, the prevalence of end stage renal diseases in China was 79.1 million in 2008^1^. There were about 270,000 registered hemodialysis (HD) patients in China in 2012, compared with only 30,000 patients receiving peritoneal dialysis^2^. Among HD patients in China, the average age was 53.8 ± 15.3 years old and the male/female ratio was 1.45:1^3^. Cardiovascular diseases (31.0%), stroke (20.3%) and infection (19.9%) were the main causes of death for HD patients in China^3^. The number of HD patients is estimated to increase rapidly in light of the aging population and the improved access to medical services in recent years^2^. A total of 3,696 HD centers were registered in China by 2011^3^, most of which were affiliated to hospitals. A set of guidance and standards for preventing infections among HD patients has been established in China since 2010^4^. However, the compliance of the prevention measures varied significantly in HD centers^4^ and a number of outbreaks of hepatitis C virus infections have been reported in China^5^.

HD patients are often in immunocompromised status and require frequent or long-term vascular access and they are therefore at risk for developing infections of the vascular access site and bloodstream infections (BSI)^6–8^. BSI and localized infections of the vascular access site cause substantial morbidity and mortality in HD patients^9, 10^. Both the American Center of Disease Prevention and Control (CDC) and the National Kidney Foundation Kidney Disease Outcomes Quality Initiative (NKF KDOQI) recommend monitoring infections in dialysis patients^11, 12^. Surveillance has long been recognized as a critical component in the prevention and control of healthcare-associated infections (HAIs)^13^. However, the infection incidence among HD outpatients in China remains unknown. The National Healthcare Safety Network (NHSN) of the CDC has developed a protocol for surveillance on several types of infection-related adverse events associated with HD called dialysis events^14^, which has the potential to be adopted or adapted for monitoring patients in China. We performed a prospective multi-center surveillance to investigate the incidence and the spectrum of dialysis events among HD outpatients in China, which is reported here.

## Methods

A prospective surveillance for dialysis events was performed for all the maintenance HD outpatients in 33 outpatient HD centers in 11 provinces in China in 2014. These centers voluntarily participated in the surveillance. There were 1,285 HD beds in total for the 33 centers with 10 to 105 beds (median, 35 beds) for each center. These centers were all affiliated to tertiary hospitals with the facility for blood culture and *in vitro* susceptibility test. Maintenance HD outpatients, including transient patients, were eligible for the surveillance if they received HD on the first two working days of the month. A total of seven dialysis events were reported and definitions for these events developed by the NHSN^14^ were applied (Table 1). Three types of dialysis events including IV antimicrobial start, positive blood culture, and pus, redness, or increased swelling at the vascular access site (Table 1) were monitored throughout the entire month. Four additional types of events, i.e. BSI, local access site infection (LASI), access-related bloodstream infection (ARB), and vascular access infection (VAI), were determined from the aforementioned three events for these patients. In addition to IV antimicrobial start, oral antimicrobial start that was not a dialysis event of NHSN was recorded in this surveillance as patients may use oral agents without prescriptions in China. Infections of a site other than BSI and LASI such as pneumonia, other respiratory tract infection and urinary tract infection were also monitored using the definitions issued by the Ministry of Health, China (Table 1)^15^. Of note, for HD patients with a BSI secondary to other infections, they were recorded as having BSI rather than other infections. For HD patients with dialysis events, admission to hospitals and deaths were recorded as the outcome. Other rules defined by NHSN such as the 21 day rule were also applied in the surveillance.

**Table 1:** Definitions of dialysis events and other infections.

Events or infections	Definitions
**Dialysis events** ^a^	
IV antimicrobial start	Any outpatient IV antibiotic and antifungal start, regardless of the reason for treatment (i.e., include IV antimicrobial starts unrelated to vascular access problems) and regardless of the duration of treatment.
Positive blood culture	Any positive blood culture collected as an outpatient or collected within 1 calendar day after a hospital admission, regardless of whether or not the patient received treatment.
Pus, redness, or increased swelling at the vascular access site	Any new outpatient episode where the patient has one or more symptoms of pus, greater than expected redness or greater than expected swelling at a vascular access site, regardless of whether the patient received treatment.
BSI	Any positive blood culture.
LASI	Pus, redness, or swelling of the vascular access site and bloodstream infection is not present.
ARB	Positive blood culture with the suspected source identified as the vascular access site or uncertain.
VAI	Either a local access site infection or an access-related bloodstream infection.
**Other infections** ^b^	
URTI	Fever (≥38.0 °C) over 2 days, plus upper respiratory tract (e.g. nasopharynx, paranasal sinus and tonsil) inflammatory manifestations.
LRTI	One of the following two: 1. Cough, sputum and moist rales plus one of the following:
	(1) Fever.
	(2) Elevated white blood cell (WBC) count and/or elevated proportion of neutrophils.
	(3) Lung inflammatory infiltrates on X-ray.
	2. Chronic respiratory tract diseases (e.g. chronic bronchitis, asthma and bronchiectasis) with acute infections, plus changes of etiology and/or new lung lesions or significant changes of original lung lesions on X-ray.
Pneumonia	LRTI plus lung inflammatory infiltrates on X-ray.
UTI	At least one of the following: urinary frequency, urinary urgency, dysuria, suprapubic tenderness, costovertebral angle pain or tenderness;
	Plus one of the following:
	(1)Urinary examination: ≥5 WBC/high power field (HPF) for male and ≥10 WBC/HPF for female.
	(2)Diagnosis of UTI by clinicians or the presence of UTI is confirmed by effective antimicrobial therapy.

^a^The definitions are from the NHSN Dialysis Event Protocol^14^.

^b^The definitions are from the standards by the Chinese Ministry of Health^15^.

Infection control link nurses in each dialysis center collected data manually from the patient chart using a self-design paper form after training and whether the HD outpatients had dialysis events or not was determined by infection control practitioners together with infection control link nurses in each HD center. The forms were input into database using EpiData (version 3.1)^16^. An auditor who was responsible for checking the quality and integrity of data and was usually an infection control practitioner other than that collected data was assigned for each participating centers. In addition, a one-month pilot study was performed on December 2013 to test the forms and the procedures before the start of the surveillance. The incidences of dialysis events were stratified by the vascular access type and were expressed by the number per 100 patient-months.

The surveillance was regarded as a performance improvement project and the ethical approval was not sought after consulting the Chinese Hospital Association. Nonetheless, the study protocol was approved by the Ethical Committee of West China Hospital, Sichuan University under a waiver of consent. This surveillance was conducted in accordance with the amended Declaration of Helsinki. Patient data were anonymized prior to analysis and investigators have no access to identifying patient information.

Statistical analysis was performed using the SPSS program (version 18.0; SPSS Inc., Chicago, IL). Percentiles were used to describe the numerical variables that fit a skewed distribution. The overall incidence of dialysis events, rate of hospitalization and mortality rate among the patients with fistula and central lines were compared using a chi square test. A *p* value below 0.05 was considered statistically significant.

## Results

During the one-year surveillance period in 2014, a total of 52,680 HD cases, which include all eligible HD patients, were monitored, corresponding to 52,680 patient months. As for the type of vascular access for HD, fistula, tunneled central line, non-tunneled central line and graft were used for 73.70% (n = 38, 824), 15.70% (n = 8,272), 8.85% (n = 4,662) and 1.04% (n = 548) of the patients, respectively. In addition, 374 (0.71%) patients received direct peripheral venipuncture of the antecubital fossa for dialysis access.

A total of 773 dialysis events occurred in 671 cases and the overall incidence of dialysis events was therefore 1.47 per 100 patient-months (773/52,680) during the surveillance period. Among the dialysis events, there were IV antimicrobial starts in 589 cases (proportion of dialysis events, 589/773, 76.20%; incidence, 1.12 per 100 patient-months), positive blood cultures in 74 (proportion, 9.57%; incidence, 0.14 per 100 patient-months) and pus, redness, or increased swelling at the vascular access site in 110 (proportion, 14.23%; incidence, 0.21 per 100 patient-months; Table 2). Among the 74 cases with BSI (positive blood cultures), 38 were ARB and the remaining 36 cases were either primary BSI without known infection sites (n = 15) or BSI secondary to other infections (n = 21; 12 cases were secondary to pneumonia, 3 to URTI, 2 to skin and sift tissue infections and one to each of LRTI other than pneumonia, liver infection, UTI and gastrointestinal infection. There were 110 cases with LASI (pus, redness, or increased swelling at the vascular access site) and the number of cases with VAI (i.e. either ARB or LASI) was therefore 148. Correspondingly, the incidences of BSI, ARB, LASI and VAI were therefore 0.14, 0.07, 0.21 and 0.28 per 100 patient-months, respectively. Among the 671 cases with dialysis events, 425 cases were admitted to hospital and the corresponding rate of hospitalization was 63.34% (425/671; Table 2). There were 12 death cases and the mortality rate was 1.79% (12/671).

**Table 2:** The incidences of dialysis events, other problems and outcomes in HD patients^a^.

Vascular access type	Patient months	Dialysis events							Other infections^b^						Hospitali-zation^c^	Death^c^
		IV antimicrobial start	BSI	LASI	ARB	VAI	Total^d^	Oral antimicrobial start	Pneumonia	Other LRTI	URTI	UTI	Others	Total		
Fistula	38,824	325 (0.84)	17 (0.04)	39 (0.10)	7 (0.02)	46 (0.12)	381 (0.98)	260 (0.67)	206 (0.53)	32 (0.08)	172 (0.44)	25 (0.06)	64 (0.16)	499 (1.29)	227, 66.76%	6, 1.76%
Graft	548	5 (0.91)	0	0	0	0	5 (0.91)	0	4 (0.73)	0	0	0	3 (0.55)	7 (1.28)	1, 20%	0
Tunneled CL^e^	8,272	152 (1.84)	35 (0.42)	40 (0.48)	15 (0.18)	55 (0.66)	227 (2.74)	73 (0.88)	60 (0.73)	16 (0.19)	32 (0.39)	7 (0.08)	36 (0.44)	151 (1.83)	115, 55.56%	4, 1.93%
Non-tunneled CL^e^	4,662	105 (2.25)	22 (0.47)	30 (0.64)	16 (0.34)	46 (0.99)	157 (3.37)	4 (0.09)	38 (0.82)	13 (0.28)	5 (0.11)	5 (0.11)	12 (0.26)	73 (1.57)	79, 68.10%	2, 1.72%
Other	374	2 (0.53)	0	1 (0.27)	0	1 (0.27)	3 (0.80)	3 (0.80)	3 (0.80)	0	5 (1.34)	1 (0.27)	1 (0.27)	10 (2.67)	3, 100%	0
Total	52,680	589 (1.12)	74 (0.14)	110 (0.21)	38 (0.07)	148 (0.28)	773 (1.47)	340 (0.65)	311 (0.59)	61 (0.12)	214 (0.41)	38 (0.07)	116 (0.22)	740 (1.40)	425, 63.34%	12, 1.79%

^a^The figures in brackets are incidences (per 100 patient-months).

^b^URTI, upper respiratory tract infection; LRTI, lower respiratory tract infection; UTI, urinary tract infection.

^c^Hospitalization and death were calculated for those with dialysis events.

^d^Total, the total number of IV antimicrobial start, BSI and LASI.

^e^CL, central line.

When stratified by the vascular access type, the incidences of dialysis events among patients using fistulas, grafts, tunneled or non-tunneled central lines, and direct puncture were 0.98, 0.91 2.74, 3.37 and 0.80 per 100 patient-months, respectively (Table 2). The dialysis events were significantly more common among patients with central lines (either a tunneled or non-tunneled) compared to those with fistulas (χ^2^, 263.198; *p*, 0.000) or graft (χ^2^, 7.935; *p*, 0.005). The incidence of dialysis events varied among hospitals from 0.00 to 16.71 per 100 patient-months (Table 3).

**Table 3:** Pool mean of the incidence (per 100 patient-months) of dialysis events in participating dialysis units.

Type of access^a^	Percentile						USA 2006	Ireland 2010^b^	Kuwait 2012^b^
	Pooled mean	10%	25%	50%	75%	90%	Pooled mean	Incidence	Incidence
IV antimicrobial start									
Fistula	0.84	0.00	0.00	0.17	1.15	3.46	1.8	1.77	5.9
Tunneled CL	1.84	0.00	0.00	0.71	4.17	7.00	6.4	NA	11.8
Non-tunneled CL	2.25	0.00	0.00	0.00	6.91	19.15	25.4	NA	11.2
BSI									
Fistula	0.02	0.00	0.00	0.00	0.00	0.18	0.5^c^	0	0.2
Tunneled CL	0.24	0.00	0.00	0.00	1.28	3.12	4.2^d^	NA	1.9
Non-tunneled CL	0.34	0.00	0.00	0.00	0.87	4.22	27.1^d^	NA	2.7
LASI									
Fistula	0.10	0.00	0.00	0.00	0.11	0.42	0.2	0	0.4
Tunneled CL	0.48	0.00	0.00	0.00	0.58	1.98	1.7	NA	2.4
Non-tunneled CL	0.64	0.00	0.00	0.00	0.04	5.05	5.1	NA	4.2
ARB									
Fistula	0.02	0.00	0.00	0.00	0.00	0.05	0.2	0	0.1
Tunneled CL	0.18	0.00	0.00	0.00	0.05	1.18	3.1	NA	1.4
Non-tunneled CL	0.34	0.00	0.00	0.00	0.16	3.43	17.8	NA	2.4
VAI									
Fistula	0.12	0.00	0.00	0.00	0.13	0.50	0.4	0	0.5
Tunneled CL	0.66	0.00	0.00	0.00	1.35	2.87	4.8	NA	3.7
Non-tunneled CL	0.99	0.00	0.00	0.00	2.01	5.71	22.9	NA	6.6

^a^The pooled means in the studies of Ireland and Kuwait are not available. NA, not available.

^b^CL, central line.

^c^The 2014 rate of BSI for fistula in USA was 0.26 (https://www.cdc.gov/nhsn/pdfs/dialysis/bsi-rate-vat-de-2014.pdf).

^d^The 2014 rate of BSI for CL (not stratified for tunneled and non-tunneled) in USA was 2.16 (https://www.cdc.gov/nhsn/pdfs/dialysis/bsi-rate-vat-de-2014.pdf).

Among 74 positive blood cultures, 57 (77.03%) isolates were recovered from patients using central lines and 17 (22.97%) were from patients with fistulas (Table 4). Of the 74 isolates from blood, 64.86% (48/74) were Gram-positive and 35.14% (26/74) were Gram-negative (Table 4). *Staphylococcus aureus* was most common microorganism, accounting for 37.84% (n = 28) of all isolates recovered. Among *S. aureus* isolates, 5 (17.86%, 5/28) were methicillin-resistant *S. aureus* (MRSA).

**Table 4:** Microorganisms recovered from blood cultures.

Microorganisms^a^	Source of positive blood culture, No. (%)		
	Central line	Fistula	Total
**Gram-positive cocci**	**34**	**12**	**46(62.16)**
Coagulase-negative staphylococci	8	2	10(13.51)
*Enterococcus faecalis*	1	0	1(1.35)
*Enterococcus faecium*	1	0	1(1.35)
*Enterococcus* spp.	1	0	1(1.35)
*S. aureus*	20	8	28(37.84)
MRSA	5	0	5(6.76)
*Streptococcus dysgalactiae*	0	1	1(1.35)
*Streptococcus gallolyticus*	0	1	1(1.35)
Species undetermined^b^	3	0	3(4.05)
**Gram-positive rods**	**2**	**0**	**2(2.70)**
*Bacillus cereus*	1	0	1(1.35)
*Corynebacterium spp.*	1	0	1(1.35)
**Gram-negative rods**	**21**	**5**	**26(35.14)**
*Alcaligenes faecalis*	0	1	1(1.35)
*Acinetobacter* spp.	3	0	3(4.05)
*Brevendimonas vesicularis*	0	2	2(2.70)
*Chromobacterium indologenes*	2	0	2(2.70)
*Enterobacter cloacae*	8	1	9(12.16)
*Escherichia coli*	2	1	3(4.05)
*Pseudomonas aeruginosa*	3	0	3(4.05)
*Pseudomonas putida*	1	0	1(1.35)
*Pseudomonas stutzeri*	1	0	1(1.35)
*Stenotrophomonas maltophilia*	1	0	1(1.35)
Total	57(77.03)	17(22.97)	74(100)

^a^Those belonged to the skin commensal microflora are underlined.

^b^The isolates were identified as Gram-positive cocci by Gram stain but the species identification was not performed.

IV antimicrobial start was the most common type of dialysis events and accounted for 76.20% (589/773) of all events. The majority (87.10%, 513/589) of IV antimicrobial start was used to treat infections other than BSI and LASI such as respiratory or urinary tract infections based on patient chart review. Most patients (72.16%, 425/589) receiving IV antimicrobial agents were admitted to hospital afterwards and the mortality rate of those receiving IV antimicrobial start was 2.04% (12/589). The majority (88.46%, 521/589) of those receiving IV antimicrobial start used a single agent. Cephalosporins, penicillins and fluoroquinolones were the most commonly-used agents with being used in 38.75%, 31.61% and 14.89% of those receiving IV antimicrobial start, respectively.

In addition, 340 HD patients received oral antimicrobial agents instead of IV ones, corresponding to 0.65 per 100 patient-months. Most (66.76%, 227/340) of oral antimicrobial agents were used to treat infections other than BSI and LASI. The hospitalization rate of those receiving oral antimicrobial agents was 20.29% (69/340), which was significantly lower than those receiving IV antimicrobial agents (72.16%; χ^2^, 232.86; *p*, 0.000). The mortality rate (0.59%; 2/340) of those receiving oral antimicrobial agents appeared to be much lower than those receiving IV antimicrobial agents (2.04%) but the difference was not statistically significant (χ^2^, 3.050; *p*, 0.081), which was likely due to the small sample size of death cases. A single agent was used in almost all (96.47%, 328/340) cases. The most commonly-used agents were cephalosporins, penicillins, macrolides and fluoroquinolones, which accounted for 40.28%, 23.66%, 16.34% and 14.37% of all use, respectively.

There were 740 patients who had an infection other than BSI and LASI with an incidence of 1.40 (740/52,680) per 100 patient-months. The majority (79.19%, 586/740) of these infections were pneumonia or other respiratory tract infections (Table 2). Other relatively common infections included urinary tract infection (n = 38), skin and soft tissue infection (n = 31), gastrointestinal infection (n = 30), and eye, ear, nose, throat, or mouth infection (n = 25).

## Discussion

To the best of our knowledge, this is the first surveillance on dialysis events in China. This surveillance generated the baseline information of infections and associated events among HD outpatients in China. In general, to counter dialysis events in China, the awareness among healthcare workers and HD patients should be raised and more efforts on prevention such as establishing a national initiative should be taken. The surveillance did generate useful information to guide the prevention in practice. First, previous studies have shown that vascular access is one of the most important risk factors for infection in HD patients^17–19^ and it has been reported that the use of fistula is associated with less incidence of complications including infections compared to the use of central lines^20^. This surveillance confirms the use of fistula for vascular access was indeed associated with lower dialysis events, hospitalization and morality than the use of central line. In light of the advantage of using fistula, it has been a core prevention measure for dialysis events. The NKF KDOQI set a goal of using fistula for vascular access in more than 65% of HD patients in 2006^21^ and subsequently the Breakthrough Initiative of Centers for Medicare and Medicaid Services (CMS) increased the goal to 66% by June 2009^22^. Although the >66% target has been achieved in the facilities participating in this study, the use of fistula should be continuously promoted and the use of central line should be minimized. Second, IV antimicrobial start accounted for the majority of dialysis events and was mainly used to treat infections other than VAI, particularly respiratory tract infections including pneumonia. Based on evidence of limited data, the intervention of dialysis events could therefore include measures to prevent respiratory tract infections such as keeping warm during the dialysis^23, 24^ and more exercises afterwards^25, 26^. Prospective interventional studies are warranted to investigate the preventability of dialysis events and the efficacy of prevention measures. Third, there were still 31 cases of ARB among those using central lines as the vascular access. Bundles targeting central line associated bloodstream infection (CLABSI) should be implemented to prevent ARB. Fourth, previous studies have identified coagulase-negative staphylococci and *S. aureus* as the major pathogens causing HD-associated infections^6, 7^. This surveillance also found that staphylococci, most of which were *S. aureus*, accounted for almost a half of all microorganisms recovered from blood cultures. However, methicillin-resistant *S. aureus* (MRSA) only accounted for 17.9% of all *S. aureus* in this surveillance, while 42% of all *S. aureus* isolates from dialysis centers participating in the NHSN were MRSA^27^. Although it still lacks large-scale and multi-center studies to screen the prevalence of MRSA in China, it appears that the carriage of MRSA was low among inpatients in this country based on several small-scale single center surveillances, e.g. 0.5% for inpatients with diabetes^28^, 1.0% for tumor patients receiving chemotherapy^29^ and 4.8% for ICU patients^30^. The relatively low prevalence of ARB due to MRSA among HD patients in this surveillance and the low carriage of MRSA among various patients in previous studies^28–30^ suggest that active screening for MRSA for HD patients may not be a cost-efficient measure in China.

Of note, the incidence of dialysis events was much lower among HD outpatients in China than those in the USA in 2006^27^, in Ireland in 2010^31^ and in Kuwait in 2012^32^ (Table 3). The incidence of BSI as a dialysis event in the USA in 2014 is also available (https://www.cdc.gov/nhsn/pdfs/dialysis/bsi-rate-vat-de-2014.pdf). The BSI incidence in the present surveillance study is still lower than that in USA in 2014 (Table 3). The exact reasons for the lower incidence of dialysis events in Chinese outpatients have not been fully understood. However, comparisons of the dialysis event incidence among different countries are complicated due to differences in the patient characteristics (e.g. underlying diseases, severity of kidney diseases, ages and personal hygiene), the access to dialysis care, staff performance in practice and the infection control policy and compliance. Nonetheless, there were several possible reasons contributing to the low incidence of dialysis events in this study. First, the utilization incidence of fistula for the vascular access among of HD patients was much higher (73.70% vs. 43%) than that in the 2006 NHSN report^27^ and as mentioned above the use of fistula is associated with lower incidence of complications. Second, the data of NHSN report on dialysis events were from 10 years ago, which is likely to be outdated. It would be more meaningful to compare our data with newer data of the NHSN but such data except those for BSI are not available yet. Third, patients with high severity of underlying or kidney diseases in China were prone to be admitted to the hospital and then receive HD during their hospitalization rather than were dialyzed in outpatients. It will be useful to perform a surveillance of dialysis events for those receiving HD during hospitalization in China. Fourth, the Ministry of Health, China, has initiated a nationwide campaign to strictly restrict the use of antimicrobial agents since May 2011, which may have significantly reduced IV antimicrobial use among HD outpatients and therefore contributes to the low incidence of dialysis events.

The low incidence of dialysis events in this study suggests that the surveillance is needed to be tailored to identify patients at high risks of infection in a resource efficient manner and therefore could be more suitable in resource-limited settings like ours. As the use of fistula was associated with lower incidences of dialysis events, we propose that the continuous surveillance of dialysis events should target those using central lines only, which are associated with higher incidence of dialysis events, more hospitalizations and increased mortality^32^. In addition, a one- or two-day point prevalence survey on dialysis events for all HD outpatients regardless of the type of vascular access could be performed several times a year, e.g. every three months, in resource-limited settings. If the point prevalence survey identified high prevalence of dialysis events in a certain type (e.g. vascular access, age and underlying diseases) of patients, targeted surveillance for those patients may be initiated.

The findings in this report are subject to several limitations and should be interpreted with cautions. First, all participating dialysis centers were in tertiary hospitals and the results in this surveillance might not be generalizable to other centers, particularly those in non-tertiary hospitals. Second, we did not specially investigate the risk factors to develop dialysis events, the interventions implemented in each center to prevent HAIs and the preventability of dialysis events due to the restrain of the manpower, material and financial resources. Third, we did not collect the data about the timing of drawing blood for culture. It was therefore likely that blood culture samples were collected after antimicrobial start, which would reduce the rate of positive cultures. Fourth, we did not monitor hospitalization and death for all HD patients but only for those with dialysis events, which was a deficiency of our study design. Therefore, we were unable to determine whether patients with dialysis events were associated with higher hospitalization and mortality rates. Nonetheless, most patients with dialysis events were admitted to hospitals, suggesting that the surveillance on dialysis events was able to identify patients with poor outcomes.

In conclusion, this first multi-site surveillance of dialysis events in China revealed a relatively low incidence of dialysis events among HD outpatients. The use of fistula was associated with lower incidence of dialysis events compared to the use of central line. IV antimicrobial start accounted for the majority of dialysis events but was mainly used to treat respiratory tract infections rather than VAI. The intervention of dialysis events could include measures to prevent respiratory tract infections in addition to those to prevent VAI including BSI and LASI. The surveillance of dialysis events may be tailored to target those using central line for vascular access in resource-limited settings.
